# Development of Phosphatized Calcium Carbonate Biominerals as Bioactive Bone Graft Substitute Materials, Part II: Functionalization with Antibacterial Silver Ions

**DOI:** 10.3390/jfb9040067

**Published:** 2018-11-23

**Authors:** Ingo Sethmann, Sabrina Völkel, Felicitas Pfeifer, Hans-Joachim Kleebe

**Affiliations:** 1 Institute of Applied Geosciences, Technische Universität Darmstadt, 64287 Darmstadt, Germany; kleebe@geo.tu-darmstadt.de; 2 Department of Biology, Technische Universität Darmstadt, 64287 Darmstadt, Germany; voelkel@bio.tu-darmstadt.de (S.V.); pfeifer@bio.tu-darmstadt.de (F.P.)

**Keywords:** bone graft substitute materials, porous calcium phosphate, coralline hydroxyapatite, phosphatized sea urchin spines, resorbable implant materials, bioactive implant materials, antibacterial properties, magnesium, strontium, silver

## Abstract

Porous calcium phosphate (CaP) materials as bone graft substitutes can be prepared from Ca carbonate biomineral structures by hydrothermal conversion into pseudomorphic CaP scaffolds. The present study aims at furnishing such phosphatized Ca carbonate biomineral (PCCB) materials with antibacterial Ag ions in order to avoid perisurgical wound infections. Prior to this study, PCCB materials with Mg and/or Sr ions incorporated for stimulating bone formation were prepared from coral skeletons and sea urchin spines as starting materials. The porous PCCB materials were treated with aqueous solutions of Ag nitrate with concentrations of 10 or 100 mmol/L, resulting in the formation of Ag phosphate nanoparticles on the sample surfaces through a replacement reaction. The materials were characterized using scanning electron microscopy (SEM) energy-dispersive X-ray spectroscopy (EDS) and X-ray diffractometry (XRD). In contact with Ringer`s solution, the Ag phosphate nanoparticles dissolved and released Ag ions with concentrations up to 0.51 mg/L, as shown by atomic absorption spectroscopy (AAS) analyses. In tests against *Pseudomonas aeruginosa* and *Staphylococcus aureus* on agar plates, antibacterial properties were similar for both types of Ag-modified PCCB materials. Concerning the antibacterial performance, the treatment with AgNO_3_ solutions with 10 mmol/L was almost as effective as with 100 mmol/L.

## 1. Introduction

Bone graft procedures are common surgical operations exceeding an annual number of two million interventions [1]. Synthetic materials as bone graft substitutes can be used to avoid drawbacks of autografts, such as additional blood loss, pain and risk of infection at the donor site [2,3,4]. Substitute materials based on CaP closely resemble human bone in composition and properties [5,6], show excellent biocompatibility and integrate well with the bone tissue [7,8]. Interconnected macropores in the implant material enhance bone regeneration and, thereby, promote the fixation of the implant [9,10,11,12,13,14,15,16]. Ideally, such an implant should actively stimulate bone formation to accelerate the osteointegration and, after stabilizing the filled defect, the implant material should become resorbed in the natural process of bone remodeling [17].

Despite their compositional similarity to bone, results of pure hydroxyapatite [HA, Ca_5_(PO_4_)_3_OH] implants were unsatisfactory, because they did not induce bone formation [18,19,20,21,22] and were hardly resorbable [23]. Implants of pure β-tricalcium phosphate [β-TCP, Ca_3_(PO_4_)_2_] with bone marrow aspirate gave better results than HA materials [21], but β-TCP was resorbed rather too fast, leaving the implant site mechanically unstable [24,25]. However, using mixtures of different CaP phases, degradation rates can be adjusted to the rate of new bone formation [26].

Among other CaP implant materials, PCCBs represent one group of materials that can be used as bone graft substitutes. Phosphatized Ca carbonate biomineral materials are based mainly of biominerals with natural macroporosity that are pseudomorphically converted into CaP materials by a coupled dissolution and precipitation process in hydrothermal phosphate solution, during which the porosity is preserved [27,28,29,30]. The most common PCCBs in clinical use are derived from coral skeletons (originally consisting of aragonite, CaCO_3_) that are converted into HA (often termed “coralline HA”) [31]. However, these materials were criticized for their lack of resorbability [32]. As a promising alternative, PCCB derived from sea urchin spines (originally consisting of calcite, CaCO_3_, with Ca ions partially substituted by Mg ions) have been tested as bone implants [29]. The substantial amounts of Mg in the reaction system shifted the phase equilibrium from HA toward the resorbable Mg- and carbonate-substituted β-TCP of which the phosphatized material mainly consisted [29,30]. Resorbable implant materials that contain Mg and Sr ions support the formation of bone and, therefore, can be considered bioactive: Mg-containing CaP enhance the adhesion, proliferation and metabolic activity of osteoblast-like cells [33]. Strontium ions stimulate the formation of bone by enhancing the osteogenic differentiation of mesenchymal stem cells [34] and additionally counteract bone loss as in osteoporosis by inhibiting bone resorption [35,36].

Many studies have been conducted on Ag-modified CaP implant materials, aiming at a decrease of the perisurgical risk of wound infections. Antibacterial properties of Ag ions have been demonstrated in studies on various materials: HA powders [37,38,39,40], HA coatings [41,42,43,44,45], HA ceramics [46,47], coralline HA [30,48], β-TCP materials [30,49,50], and mixed phase CaP ceramics [51,52]. The antimicrobial effect is directly related to the release of Ag ions which inhibit the proper functioning of the bacterial ribosome [53] and have an adverse effect on bacterial DNA replication [54]. In moderate concentrations, Ag ions can be antimicrobially effective without impairing the cytocompatibility [47,55]. Generally, microbial growth is inhibited in the presence of metallic, colloidal or ionic Ag [30,56,57].

Part I of the present study was aimed at developing coral and sea urchin-derived PCCB towards resorbable, bioactive and antiosteoporotic bone graft substitute materials by the incorporation of Mg and/or Sr ions [58]. Part II, presented here, is a feasibility study dedicated to endowing the resulting materials with antibacterial properties by adding Ag ion-releasing nanoparticles to the material surfaces. It was intended to investigate the replacement reaction system of Mg- and/or Sr-modified porous PCCB in Ag nitrate solutions for the composition and distribution of the Ag-containing phases produced as well as their properties in releasing Ag ions upon dissolution in Ringer’s solution and their antibacterial effectiveness, respectively.

## 2. Results and Discussion

### 2.1. Modification with Silver Ions

The coral-derived starting material consisted of Na- and Sr-doped HA combined with Sr-substituted β-TCP [(Ca,Sr)_3_(PO_4_)_2_] as a surface layer and some aragonite. The sea urchin-derived starting material was composed of merrillite (Na-Mg-substituted β-TCP; Ca_9_NaMg(PO_4_)_7_) with some additional Sr as the dominant phase, HA as a secondary phase and some Mg-calcite.

**Figure 1:**
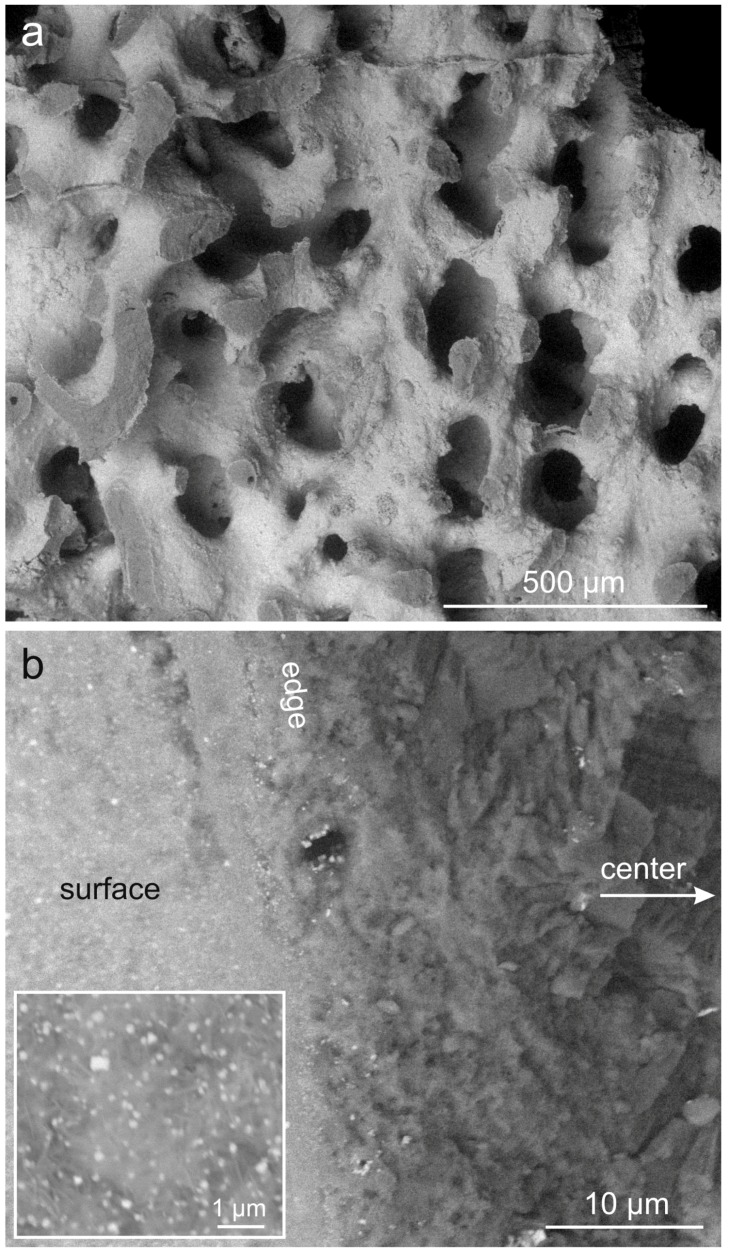

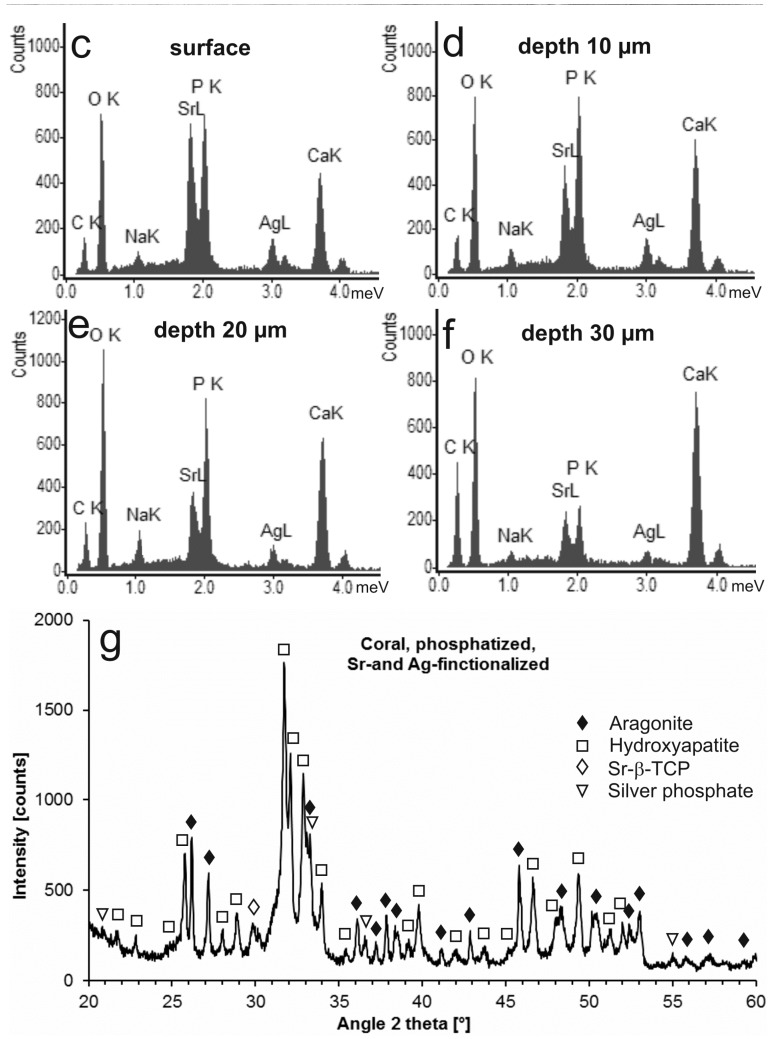
Phosphatized and Sr-modified coral skeleton with additional Ag functionalization (sample C Ag 100). (**a**) Porous structure of the material (environmental scanning electron microscopy-backscatter electron (ESEM-BSE)); (**b**) a fractured trabecula (fracture edge indicated) showing the internal microstructure of the material on the right hand side (darker shading; center of trabecula indicated) and the material surface (indicated) with a layer of small crystals composed of heavier elements (lighter shading) on the left hand side and dispersed nanocrystals composed of heavy elements (white dots; see inset) (ESEM-BSE). Energy-dispersive X-ray spectroscopy analyses show the chemical composition at (**c**) the surface and of the interior of the material at depths of (**d**) 10 µm, (**e**) 20 µm, and (**f)** 30 µm. (**g**) Crystalline phases contained in the material, identified by XRD (aragonite PDF 00-041-1475, hydroxyapatite PDF 00-009-0432, Sr-β-TCP, c.f. [59], Ag phosphate PDF 00-006-0505); XRD raw data S1: C33.

Phosphatized and Mg and/or Sr-modified materials were treated with Ag nitrate solutions with concentrations of either 10 mmol/L (sample codes C Ag 10 for coral- and SU Ag 10 for sea urchin-derived materials) or 100 mmol/L (sample codes C Ag 100 for coral- and SU Ag 100 for sea urchin-derived materials). This treatment did not affect the porous structure of the materials as visible for the coral-derived material in Figure 1a. The reaction resulted in the precipitation of nanoparticles evenly dispersed at the surfaces of the samples (Figure 1b and inset). Here, the difference between the samples C Ag 100 (Figure 1) and C Ag 10 consisted in a much lower density of particles in the latter case (Figure S1). Element analyses with environmental scanning electron microscopy (ESEM)-based energy-dispersive X-ray spectroscopy (EDS) were done at fractured trabeculae in steps of 10 µm from the surface to the center (Figure 1c–f). Energy-dispersive X-ray spectroscopy analyses indicated that the pseudomorphic phosphate phase contained a constant concentration of C (Figure 1c–e). Since in this case the sample was not coated with carbon, this C content may represent carbonate ions originating from dissolution of aragonite and reprecipitating as substituents in the newly formed CaP phase. However, the analyzed carbon content could also represent carbon from the atmosphere fixed on the sample surface. Nevertheless, an incorporation of some carbonate into the pseudomorphic CaP phases has to be expected for all described experiments of phosphatization. At the center of trabecula, some original aragonite persisted as indicated by the higher carbon content (Figure 1f; mixed analysis of original aragonite and pseudomorphic phosphate material). At the surface, substantial concentrations of Ag were detected (Figure 1c), which can be correlated with the nanoparticles visible in Figure 1b. Even below the surface, Ag was detected with lower concentrations in the internal phosphate material (Figure 1d–f). Determination of the crystalline phases in the material by means of XRD yielded a residue of the original aragonite, HA as the predominant pseudomorphic phase, small amounts of Sr-β-TCP, c.f. [59] (probably the main phase at the surfaces), and Ag phosphate (Ag_3_PO_4_; PDF 00-006-0505) (Figure 1g).

**Figure 2:**
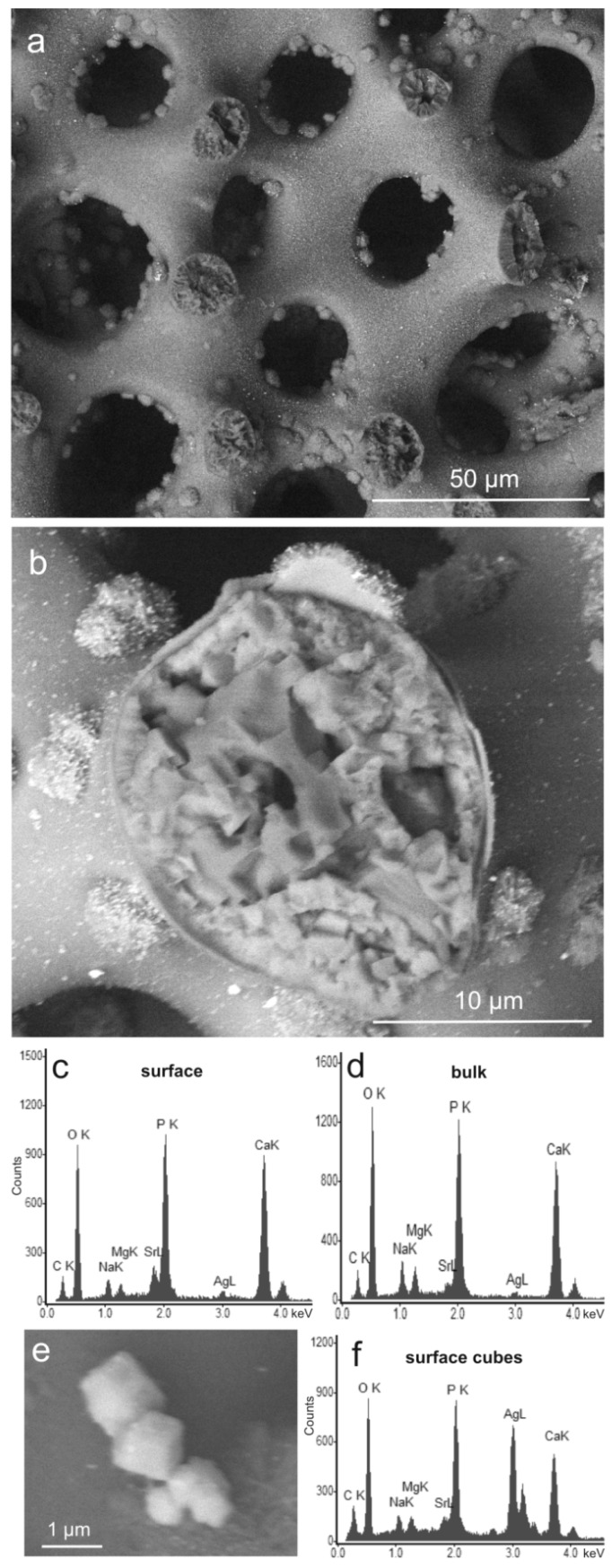

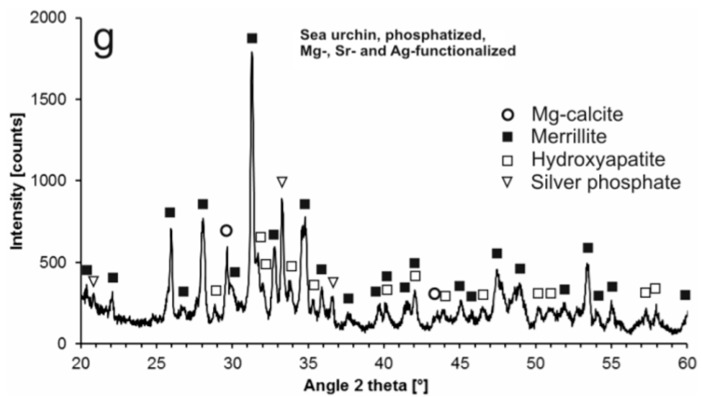
Phosphatized and Sr-modified sea urchin spine with additional Ag functionalization (sample SU Ag 100). (**a**) Porous structure of the material (ESEM-BSE); (**b**) a fractured trabecula showing the internal microstructure of the material and rose-shaped aggregates of crystals grown on the surface with additional microcrystals composed of heavy elements dispersed on the surface (white dots; ESEM-BSE). Energy-dispersive X-ray spectroscopy analyses show the chemical composition of (**c**) the surface material and (**d**) the bulk material. (**e**) Cubic microcrystals on the material’s surface. (**f**) chemical composition of these microcrystals (EDS). (**g**) Mineral phases contained in the material, identified by XRD (Mg-calcite PDF 00-043-0697, merrillite PDF 01-076-8368, hydroxyapatite PDF 00-009-0432, and Ag phosphate PDF 00-006-0505); XRD raw data S2: SU25.

In the case of sea urchin-derived phosphatized and Sr-modified material, treatment with Ag nitrate solution did not affect the porous morphology of the sample (Figure 2a) but resulted in nanoparticles dispersed at the sample surface (Figure 2b), similar to those of coral-derived material. Correspondingly, Ag was detected at the surface of the sample (Figure 2c) and also in the bulk material (Figure 2d). Larger specimens of the surface crystals displayed distinct cubic morphologies (Figure 2e) and were shown to contain high concentrations of Ag (Figure 2f). Beside Ag, Sr, Mg, and Na, the CaP material contained some C as well (Figure 2c,d). This C content probably represents at least partly carbonate ions dissolved from the sea urchin calcite along with the Ca and Mg ions, which subsequently reprecipitated as substituted CaP. Here as well, the detected carbon may also represent carbon from the atmosphere fixed on the sample surface. In addition to the crystalline phases of the material before Ag functionalization took place (Mg-calcite, merrillite, and HA), the XRD pattern shows distinct peaks of Ag phosphate (Figure 2g), which corresponds to the crystals with cubic morphology in Figure 2e and the chemical composition shown in Figure 2f.

Treating the Sr-modified phosphate materials with an aqueous solution of Ag nitrate produced Ag phosphate at the surfaces, the vast majority in the form of nanoparticles, but interspersed with some microparticles. The reason for the growth of these few larger particles could not be identified. The precipitation of Ag phosphate was coupled with the dissolution of Sr-β-TCP or Sr-merrillite, respectively, according to the following simplified reactions:

Ca_3_Sr_3_(PO_4_)_4_ + 12 Ag^+^ ⟹ 4 Ag_3_PO_4_ + 3 Ca^2+^ + 3 Sr^2+^ (1)

or

Ca_8_NaMgSr(PO_4_)_7_ + 21 Ag^+^ ⟹ 7 Ag_3_PO_4_ + 8 Ca^2+^ + Na^+^ + Mg^2+^ + Sr^2+^ (2)

Compared to their small volume, nanoparticles have large surface areas at which reactions with fluid environments can take place. Therefore, the small size of Ag phosphate particles and their even distribution at the surfaces are promising material properties for rapid and spatially uniform dissolution and release of Ag ions upon application. In another study on coralline HA as potential bone implant material, a similar approach was found to be viable for an effective antibacterial Ag-modification of the material [48].

### 2.2. Release of Silver Ions upon Dissolution

The antibacterial effect of Ag in bone graft substitute materials is assumed to be based mainly on the release of Ag ions upon dissolution of Ag-containing compounds in the surrounding body fluid, which starts after the implant surgery. In an experimental approach to this process, coral- and sea urchin-derived samples with Ag-functionalization (samples C Ag 100 and SU Ag 100) were held in Ringer’s solution at body temperature. The aim of these experiments was to demonstrate that Ag ions actually become released from these materials and to determine the concentration range for Ag ions in solution. For comparison, samples of Ag phosphate and Ag chloride were treated in the same way, because these were the relevant Ag compounds in the reaction systems. The solutions resulting from these experiments as well as a sample of the pristine solvent (sample Ringer) were analyzed using atomic absorption spectroscopy (AAS) (Table 1). For coral- and sea urchin-derived materials, concentrations of dissolved Ag ions were found to be in the range of 0.45 to 0.51 mg/L. The solubilities of Ag phosphate and Ag chloride in Ringer’s solution were both found to be in a similar range, while Ringer’s solution itself contained only negligible concentrations of Ag ions. The concentrations found for the Ag ions did not clearly show dependence of the reaction time (24 h or 72 h) with Ringer’s solution, which presumably indicated that the solutions were already nearly saturated with respect to the relevant substances within 24 h. Non-systematic variations in the solubility or dissolution rates may be caused by differences in the materials’ crystallinity or particle sizes.

The incorporation of Ag ions into the materials was intended to provide the materials with antibacterial properties upon implantation. The narrow range of Ag ion concentrations released into Ringer’s solution by all samples, largely independent of the reaction time after 24 h (Table 1), reflect fast initial dissolution kinetics of the Ag phosphate nanoparticles at the material surfaces. This dissolution process is coupled with reprecipitation of Ag ions as Ag chloride, which is the least soluble salt of Ag in this environment. Therefore, the concentration of dissolved Ag ions is limited by the solubility of Ag chloride. According to a thermodynamic calculation with Visual MINTEQ 3.1 [60], the theoretical solubility of Ag chloride in Ringer’s solution at 37 °C is 12.72 µmol/L, equaling a weight-based concentration of Ag ions of about 1.37 mg/L. In simulated serum electrolyte solution, the maximum concentration of Ag ions is even lower at about 0.15 mg/L [61]. These measured and calculated values suggest that dissolution of Ag salts in chloride-containing solutions will usually produce Ag ion concentrations exceeding the threshold for antibacterial effectiveness (≥0.1 mg/L) but staying below cytotoxic levels (≥1.6 mg/L) [55]. Nevertheless, upon implantation, all Ag salts present will become completely dissolved in serum over time, because the surrounding tissue and body fluid represent a semi-open system in which dissolved species disperse and hence become diluted continuously. In this system, gradual dissolution of Ag salts and dispersion of Ag ions are additionally promoted by serum proteins which sequester these ions [61], acting as a mobile temporary sink for Ag ions and facilitate their dispersion. The total amount of Ag ions in implants should, however, be limited to the minimum required for antibacterial effectiveness, because finally they tend to accumulate in the liver and other organs and may impair their functions [61].

### 2.3. Antibacterial Tests

Antibacterial properties of Ag-functionalized samples were determined by means of inhibition of two different bacterial strains, *Pseudomonas aeruginosa* and *Staphylococcus aureus*. Bacterial inhibition was expected as an effect of Ag ions which dissolved from the samples and diffused through the agar gel substrate. In these experiments, the width of the area of inhibition served as a measure of antibacterial effectiveness.

**Figure 3:**
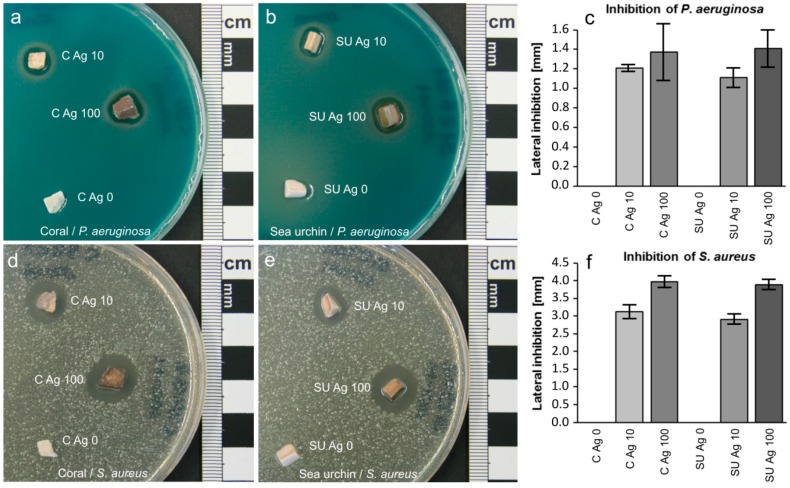
Antibacterial effects of phosphatized and Sr-modified materials with and without additional Ag functionalization on bacterial strains of *Pseudomonas aeruginosa* (Gram-negative) and *Staphylococcus aureus* (Gram-positive). *Pseudomonas aeruginosa*-seeded agar plates inoculated with (**a**) coral-derived materials and (**b**) sea urchin-derived materials and (**c**) measurements (with standard deviations) of the lateral bacterial inhibition areas for the different materials. *Staphylococcus aureus*-seeded agar plates inoculated with (**d**) coral-derived materials and (**e**) sea urchin-derived materials and (**f**) measurements (with standard deviations) of the lateral bacterial inhibition areas for the different materials. (C Ag 0 and SU Ag 0: no treatment with Ag ions; C Ag 10 and SU Ag 10: treated with 10 mM Ag solution; C Ag 100 and SU Ag 100: treated with 100 mM Ag solution.)

As expected, all Ag-functionalized samples clearly showed antibacterial properties. In the case of Gram-negative *P. aeruginosa*, the area of inhibition was about 1.1–1.2 mm wide with C Ag 10 and SU Ag 10 samples being insignificantly less effective than C Ag 100 and SU Ag 100 samples (Figure 3a–c). Gram-positive *S. aureus* was more sensitive to Ag ions. Here, C Ag 10 and SU Ag 10 samples effected inhibition areas with widths of about 3 mm, while C Ag 100 and SU Ag 100 samples were slightly more effective with about 4 mm width of the inhibition areas (Figure 3d–f). Generally, coral- and sea urchin-derived materials with corresponding Ag ion-treatment did not show significant differences in their antibiotic effectiveness. Ag-free control samples C Ag 0 and SU Ag 0 with no antibacterial effect clearly indicate that the antibacterial properties of C Ag 10 and SU Ag 10 samples as well as those of C Ag 100 and SU Ag 100 samples are based on their Ag content and not on other components of the samples.

As demonstrated by the results of the dissolution experiments (Table 1), Ag phosphate particles dissolve in a physiological fluid and release Ag ions. The antibacterial tests presented here are based on the dispersion of released Ag ions in the surrounding agar medium by diffusive transport. The maximum distance at which an inhibition of bacterial growth was observed (i.e., the diffusion front with a minimum concentration of Ag ions for an antibacterial effect) was used as a measure of the antibacterial effectiveness. This approach was already chosen in earlier studies [30,48]. In our antibacterial tests, the larger areas of inhibition for Gram-positive *S. aureus* suggest lower concentrations of Ag ions to be sufficient in this case compared to *P. aeruginosa* (Gram-negative) exhibiting smaller inhibition areas. In turn, it follows that *S. aureus* is more sensitive to the antibacterial effect of Ag ions than *P. aeruginosa*. This finding of differential sensitivity is in accordance with the finding of Schlosser et al. [30] and was also observed in corresponding experiments with the antibiotic ampicillin (Figure S2). The higher level of resistance of *P. aeruginosa* is based on the presence of an additional outer membrane in Gram-negative bacteria [62]. In Ag-based inhibition experiments, the samples with lower contents of Ag phosphate (C Ag 10 and SU Ag 10) were generally equally or nearly as effective against bacterial growth as samples with considerably higher Ag contents (C Ag 100 and SU Ag 100). This finding agrees with observations by Zhang et al. [48] and may suggest the resource-conserving use of lower concentrated Ag nitrate (10 mmol/L) solution during antibacterial functionalization, which also reduces the total load of potentially adverse Ag ions introduced to the organism. Small amounts of surface-attached Ag phosphate releasing Ag ions over a time period of only a few days should be sufficient for the desired antibacterial effect, because an acute risk of infection exists mainly during the surgery and shortly thereafter. Hence, a long-term antibacterial functionality is not necessary. With regard to a possible future application as implant materials, however, it seems difficult to estimate the amount of Ag phosphate necessary to ensure the persistence of a sufficient reservoir for Ag ions over a period of a few days. Trials with more realistic model systems would be mandatory.

## 3. Materials and Methods

### 3.1. Starting Materials

The starting materials for the present study were prepared and characterized during previous research work [58]: A coral skeleton (*Porites* sp., aragonite, CaCO_3_) and a sea urchin spine (*Heterocentrotus mamillatus*, Mg-calcite, Ca_9_Mg(CO_3_)_10_) were cut to produce samples of about 0.5 to 0.7 g. These samples were cleaned of organic material with hydrogen peroxide solution (H_2_O_2_ 10%) and subjected to hydrothermal treatment with an aqueous solution of Na_2_EDTA · H_2_O (0.2 mol/L, ethylenediaminetetraacetic acid disodium salt dihydrate), SrCl_2_ · 6 H_2_O (0.1 mol/L), and Na_2_HPO_4_ · 2 H_2_O (1.0 mol/L). Each sample was placed in 35 mL solution in a Teflon^TM^-lined autoclave (steel bomb, 125 mL volume; Parr Instrument, Frankfurt, Germany) and held at 200 °C for 168 h. The porous Ca carbonate biominerals were partially converted into pseudomorphic PCCB via a coupled dissolution and precipitation mechanism with additional Na and Sr ions from the solution incorporated in the CaP phases. The phosphatized sea urchin material also contained substantial concentrations of Mg ions that were reprecipitated together with the Ca ions after Mg-calcite dissolution. At the start of the present study, the coral-derived material consisted of microcrystalline, Na- and Sr-doped HA with a surface layer of Sr-substituted β-TCP [(Ca,Sr)_3_(PO_4_)_2_] beside residual aragonite. In the case of the sea urchin-derived material, the Mg ions induced a shift towards the formation of merrillite (Na-Mg-substituted β-TCP; Ca_9_NaMg(PO_4_)_7_) with an additional Sr content as the dominant phase, while HA occurred as a secondary but still significant phase beside residual Mg-calcite. The surfaces of the sea urchin-derived material were studded with rose-shaped crystal aggregates probably consisting of Sr-substituted β-TCP.

### 3.2. Treatment with Silver Ions

The samples were held in aqueous solutions of AgNO_3_ (10 mmol/L or 100 mmol/L) at 25 °C for 24 h (sample codes in Figure 3: C Ag 10 and C Ag 100 for coral materials; SU Ag 10 and SU Ag 100 for sea urchin materials). Afterwards, they were thoroughly washed in deionized water and dried using a tissue that absorbed the pore fluid to avoid precipitation due to evaporation. The entire treatment, storage and characterization of these samples were performed under ruby light or at low light intensity, because a light-sensitive phase was expected to precipitate during the interface reaction.

### 3.3. Sample Characterization

The samples were cleaved to expose fresh fracture surfaces of the material before mounting the samples on aluminum stubs with carbon tabs for characterization with scanning electron microscopy (SEM; FEI ESEM Quanta 200 FEG, ThermoFisher Scientific, Hillsboro, OR, USA) at 12.5 kV accelerating voltage. The Ag-modified samples remained uncoated, because the light-emitting carbon-coating process would possibly alter light-sensitive phase in samples. Hence, the SEM was operated in low-vacuum mode with an atmosphere of 1.3 mbar water vapor in the sample chamber (environmental scanning electron microscopy, ESEM), which provided electron conductivity at the cost of slightly reduced image resolution. The morphologies of the samples and material contrasts were imaged in backscatter electron (BSE) imaging mode. Additionally, the chemical compositions of the sample materials were determined using SEM-integrated energy-dispersive X-ray spectroscopy (EDS; EDAX, Mahwah, NJ, USA). Here, the uncoated samples allowed for unbiased analyses of the carbon contents.

For identification of the crystalline phases, selected samples were powdered in an agate mortar prior to applying X-ray diffraction (XRD; D8, Bruker, Billerica, MA, USA) in Bragg–Brentano geometry using Cu*K_α_* radiation. Sample C Ag 100 was consistent with XRD sample C33 (XRD raw data S1), while sample SU Ag 100 was consistent with XRD sample SU25 (XRD raw data S2).

### 3.4. Cation Release upon Dissolution

The release of Ag ions upon the reaction of the samples with Ringer’s solution (an aqueous solution containing NaCl (8.6 g/L), KCl (0.3 g/L), and CaCl_2_ · 2 H_2_O (0.33 g/L) at pH 7.4, isotonic in relation to body fluids), was investigated. Cleaved pieces of about 50 mg of the samples C Ag 100 and SU Ag 100 were held light-protected in 20 mL of Ringer’s solution at 37 °C for 24 h and 72 h, respectively. Samples of 10 mg of AgCl and Ag_3_PO_4_, respectively, held in Ringer’s solution in the same way as well as pure Ringer’s solution served as controls. The resulting solutions were analyzed for Ag ions using flame atomic absorption spectroscopy (AAS; contrAA 300, Analytik Jena, Jena, Germany). The detection limit for Ag^+^ was 0.02 mg/L.

### 3.5. Antibacterial Tests

Antibacterial properties of the Ag-modified samples were tested against two bacterial strains relevant for wound infections, *Staphyloccocus aureus* DSM 1104 (Gram-positive) and *Pseudomonas aeruginosa* DSM 1117 (Gram-negative). Both strains were cultivated in yeast extract dextrose medium (YED; 5 g/L peptone, 2.5 g/L yeast extract, 1 g/L glucose, pH 7.0) at 37 °C and 180 rpm. Solid media were prepared with YED and agar (15 g/L), top layered with seeded soft agar of YED with agar (10 g/L), containing a known number of bacteria (10^8^ cfu/mL). Three different cleaved samples of either coral- or sea urchin-derived materials were added to each agar plate for direct comparison: two samples modified in solutions of AgNO_3_ and one sample without Ag-modification: C Ag 10, C Ag 100 and C Ag 0 or SU Ag 10, SU Ag 100 and SU Ag 0 (each sample about 50 mg). These tests were carried out in triplets for each type of materials, coral- and sea urchin-derived CaP scaffolds, in combination with each bacterial strand, respectively (12 separate test plates in total). As a control and for comparison, for each bacterium one plate was prepared with two filter papers soaked with the antibiotic ampicillin (1 mg/mL and 100 mg/mL, respectively). Inoculated plates were pre-incubated at 4 °C for 2 h prior to incubation at 37 °C for 24 h. After this incubation time, the zones of inhibition were measured at the agar surface radially from the sample rim in five directions per sample. From these 15 measurements per sample type (triplet), the arithmetic mean and the standard deviation were calculated. Except for a few modifications, partly inspired by Zhang et al. [48], the described procedure follows the protocol of Schlosser et al. [30].

## 4. Conclusions

In a previous study, phosphatized calcium carbonate biominerals, specifically coral- and sea urchin-derived CaP scaffolds, were developed into porous bone graft substitute materials with bioactive functionality through incorporation of Mg and/or Sr ions [58]. The resulting materials were essentially triphasic, the coral material consisting of HA, Sr-substituted β-TCP, and aragonite, while the sea urchin-derived material was composed of Sr-substituted merrillite (Na-Mg-(Sr)-β-TCP), HA and Mg-calcite.

The present study demonstrates the treatment of these materials with Ag nitrate solutions to precipitate Ag phosphate nanoparticles on the surfaces of the samples which, upon dissolution, release Ag ions. Due to a self-limiting property of the reaction system in body fluid, the concentrations of dissolved Ag ions are expected to remain in a range above the threshold for antibacterial effectiveness and below cytotoxicity. Therefore, the Mg- and/or Sr-modified PCCB materials, previously developed for potential osteoinductive bioactivity, were further functionalized for reducing the risk of perisurgical wound infections during possible future application as bone graft substitutes. The load of Ag phosphate in the samples could be controlled by using different concentrations of Ag nitrate for the treatment of the starting materials. In our system, samples treated with an AgNO_3_ solution with 10 mmol/L showed nearly the same antibacterial performance as samples treated with 100 mmol/L. It is difficult to estimate the optimum load of Ag_3_PO_4_ in the materials for an application as implants: on the one hand, Ag phosphate content should provide a sufficient source for Ag ion release for an antibacterial effect over a few days. On the other hand, the total load of Ag ions should be restricted to a minimum to avoid health problems [61]. Trials with more realistic model systems are required optimizing the load of Ag phosphate.

## Figures and Tables

**Table 1:** Silver ion concentrations determined with atomic absorption spectroscopy (AAS) after material dissolution in Ringer’s solution at 37 °C for 24 h and 72 h.

Sample	Reaction Time	Ag^+^ (mg/L)
C Ag 100	24 h	0.45
C Ag 100	72 h	0.51
SU Ag 100	24 h	0.51
SU Ag 100	72 h	0.47
Ag_3_PO_4_	24 h	0.49
Ag_3_PO_4_	72 h	0.59
AgCl	24 h	0.46
AgCl	72 h	0.46
Ringer	--------	0.06

## Supplementary Materials

The following are available online at http://www.mdpi.com/2079-4983/9/4/67/s1, Figure S1: Distribution of Ag phosphate nanoparticles on sample surfaces; Figure S2: Effect of ampicillin on *Pseudomonas aeruginosa* and *Staphylococcus aureus*; XRD raw data S1: C33; XRD raw data S2: SU25.
