# Supplementary material for: Development of Phosphatized Calcium Carbonate Biominerals as Bioactive Bone Graft Substitute Materials, Part II: Functionalization with Antibacterial Silver Ions
Source: J Funct Biomater. 2018 Nov 23;9(4):67. doi: 10.3390/jfb9040067 (PMC6306760; doi:10.3390/jfb9040067)
Supplement: Supplementary file 1 [file jfb-09-00067-s001.zip › Figure S1.pdf]

Supplementary material: Figure S1

Development of phosphatized calcium carbonate biominerals as bioactive bone graft substitute materials, part II: functionalization with antibacterial Ag ions

Ingo Sethmann [1,*], Sabrina Völkel [2], Felicitas Pfeifer [2], Hans-Joachim Kleebe [1]

[1] Institute of Applied Geosciences, Technische Universität Darmstadt, 64287 Darmstadt, Germany

[2] Department of Biology, Technische Universität Darmstadt, 64287 Darmstadt, Germany

* Correspondence: ingo.sethmann@gmail.com

Distribution of Ag phosphate nanoparticles on sample surfaces

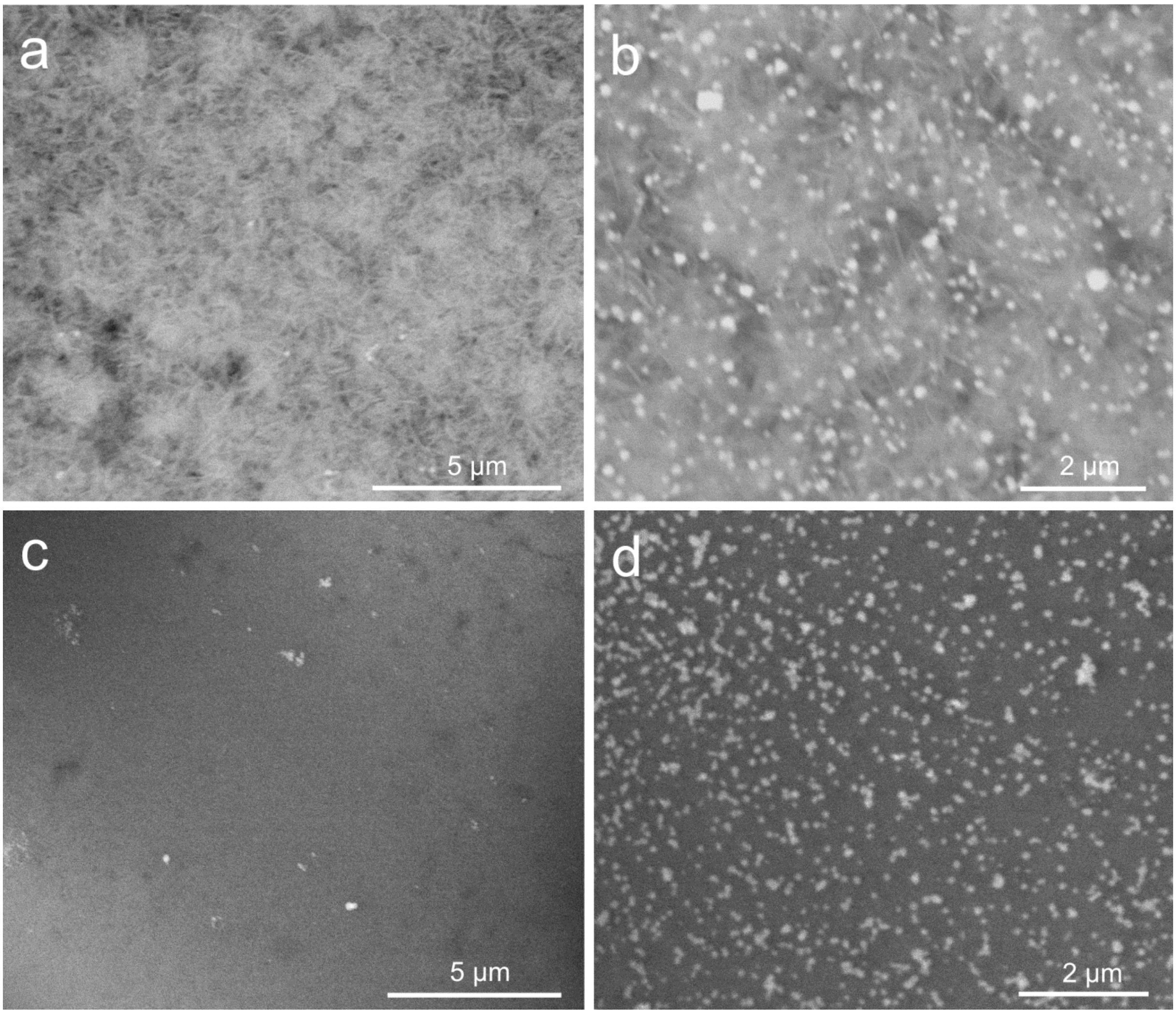

Figure S1. Ag phosphate nanoparticles (white dots) on the surfaces of coral-derived material treated with (**a**) 10 mM Ag nitrate solution and (**b**) 100 mM Ag nitrate solution and on the surfaces of sea urchin-derived material treated with (**c**) 10 mM Ag nitrate solution and (**d**) 100 mM Ag nitrate solution; environmental scanning electron microscopy (ESEM) images.
