# Supplementary material for: Development of Phosphatized Calcium Carbonate Biominerals as Bioactive Bone Graft Substitute Materials, Part II: Functionalization with Antibacterial Silver Ions
Source: J Funct Biomater. 2018 Nov 23;9(4):67. doi: 10.3390/jfb9040067 (PMC6306760; doi:10.3390/jfb9040067)
Supplement: Supplementary file 1 [file jfb-09-00067-s001.zip › Figure S2.pdf]

Supplementary material: Figure S2

Development of phosphatized calcium carbonate biominerals as bioactive bone graft substitute materials, part II: functionalization with antibacterial Ag ions

Ingo Sethmann [1,*], Sabrina Völkel [2], Felicitas Pfeifer [2], Hans-Joachim Kleebe [1]

[1] Institute of Applied Geosciences, Technische Universität Darmstadt, 64287 Darmstadt, Germany

[2] Department of Biology, Technische Universität Darmstadt, 64287 Darmstadt, Germany

* Correspondence: ingo.sethmann@gmail.com

Effect of ampicillin on *Pseudomonas aeruginosa* and *Staphylococcus aureus*

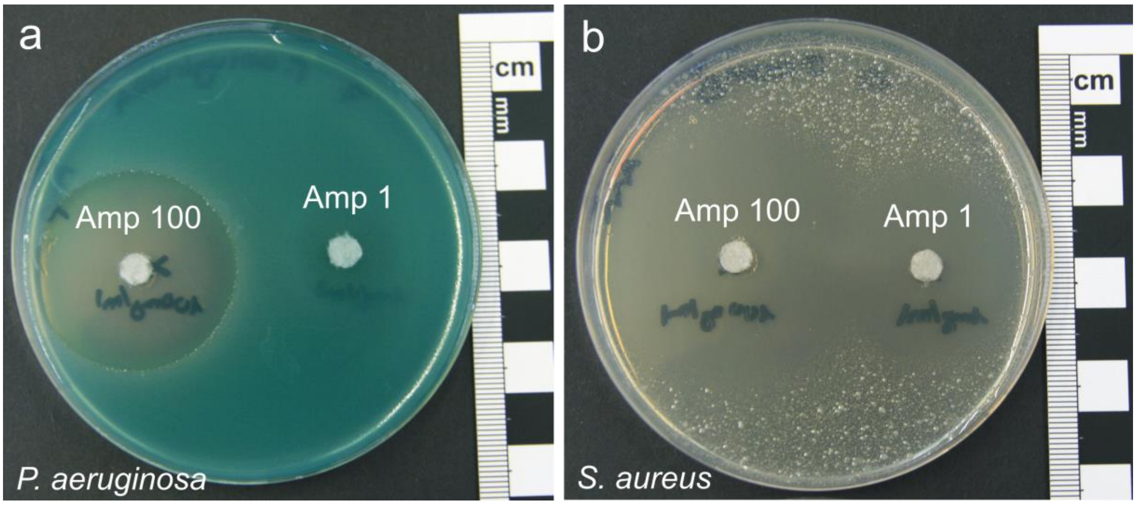

Figure S2. Antibacterial effects of the antibiotic ampicillin on bacterial strains of (**a**) *Pseudomonas aeruginosa* (Gram-negative) and (**b**) *Staphylococcus aureus* (Gram-positive). [Amp 1: ampicillin 1 mg/ml; Amp 100: ampicillin 100mg/ml]
